# First description of the male of *Barylestissaaristoi* Jäger, 2008 (Araneae, Sparassidae) from China

**DOI:** 10.3897/zookeys.832.32569

**Published:** 2019-03-20

**Authors:** Yang Zhong, Ye-Jie Lin, Jie Liu

**Affiliations:** 1 School of Nuclear Technology and Chemistry & Biology, H; 2 ubei University of Science and Technology, Xianning 437100, Hubei, China; 3 The State Key Laboratory of Biocatalysis and Enzyme Engineering of China, Centre for Behavioural Ecology and Evolution, College of Life Sciences, Hubei University, Wuhan 430062, Hubei, China; 4 Institute of Zoology, Chinese Academy of Sciences, Beijing 100101, China

**Keywords:** biodiversity, Yunnan, huntsman spiders, taxonomy

## Abstract

The male of *Barylestissaaristoi* Jäger, 2008 is described for the first time from Menglun Town, Yunnan Province, China. This is the first record of this genus from China. An illustrated morphological description of this species is given.

## Introduction

The genus *Barylestis* was established by Simon (1910). Currently, of all 11 known *Barylestis* species, 10 from tropical Africa (Cameroon, Congo, Central Africa, Equatorial Guinea, Gabon, Nigeria, Rwanda, Sudan, Uganda, West Africa) and one from Southeast Asia (Thailand, Myanmar) have been recorded (Jäger 2002; World Spider Catalog 2018). This genus had long been recognized as a purely African genus by Jäger (2002) until *Barylestissaaristoi* Jäger, 2008 was first described based on female specimens from Thailand and Myanmar. Jäger and his colleagues tried to collect the male of *B.saaristoi* specifically because of its potential contribution to zoogeographic and phylogenetic relationships of this genus, but the search was not successful (Jäger 2008). Recently, the authors examined specimens collected from Yunnan Province and found three females and one male belonging to this species. This is also the first record of this genus from China.

## Materials and methods

Specimens were examined and measured with a Leica M205C stereomicroscope. Epigynes were examined and illustrated after dissection from the spider bodies. All photos were taken with a Leica DFC450 digital camera attached to a Leica M205C stereomicroscope, with 10–20 photographs taken in different focal planes and combined using image stacking software (Leica LAS). Photographic images were edited using Adobe Photoshop. Left palps are depicted. Most hairs and macrosetae are omitted in the palp drawings. All specimens examined in this study are deposited in the College of Life Sciences, Hubei University.

Leg measurements are shown as: total length (femur, patella, tibia, metatarsus, tarsus). Number of spines is listed for each segment in the following order: prolateral, dorsal, retrolateral, ventral (in femora and patellae ventral spines are absent and the fourth digit is omitted in the spination formula). Abbreviations follow Zhong et al. (2017, 2018):

**ALE** anterior lateral eyes;

**AME** anterior median eyes;

**AW** anterior width of prosoma;

**CH** clypeus height;

**FE** femur;

**Mt** metatarsus;

**OL** opisthosoma length;

**OW** opisthosoma width;

**Pa** patella;

**PL** prosoma length;

**PLE** posterior lateral eyes;

**PME** posterior median eyes;

**Pp** palp;

**PW** prosoma width;

**Ta** tarsus;

**Ti** tibia I, II, III, IV—legs I to IV.

Abbreviations for the collection depositories:

**HBU**Hubei University, Wuhan, China;

**SMF** Research Institute Senckenberg, Frankfurt, Germany

## Taxonomy

### Family Sparassidae Bertkau, 1872

#### Genus *Barylestis* Simon, 1910

##### Barylestis saaristoi

Taxon classificationAnimaliaAraneaeSparassidae

Jäger, 2008

Figures 1–3 , 4–8 , 9–21 , 22–25 , 26

Barylestis saaristoi Jäger, 2008: 106, figs 1–14 (holotype female from Mae Hong Son Province, Thailand, deposited in SMF 58342).

###### Material examined.

1 male and 1 female (HBU), Mengxin Farm [21.89°N, 101.36°E, 736m], Dai Autonomous Prefecture of Xishuangbanna, China, 4 May 2018, Yiwu Zhu leg.; 2 females (HBU), Xishuangbanna Tropical Botanical Garden [21.96°N, 101.22°E, 757m] Dai Autonomous Prefecture of Xishuangbanna, China, 30 May 2015, Wancheng Li leg.

###### Diagnosis.

Male of *B.saaristoi* can be separated from *B.montandoni* (Lessert, 1929) and *B.occidentalis* (Simon, 1887) by embolus arising from tegulum in an 11-o’clock-position (3-o’clock-position in *B.montandoni* and *B.occidentalis*, Figs 2, 5), separated from *B.fagei* (Lessert, 1929) and *B.variatus* (Pocock, 1900) by the long and slender dRNA (short and wide in *B.fagei* and *B.variatus*, Figs 3, 6), separated from *B.scutatus* (Pocock, 1903) by tegulum partly covered embolic base (wholly covered in *B.scutatus*). Females of this species can be recognised as this particular species by the following combination of characters: 1. Epigyne with V-shaped pit; 2. Vulva with tips of lateral coils pointing mediad and first part of copulatory ducts slender, running parallel (Jäger 2008).

**Figures 1–3.**
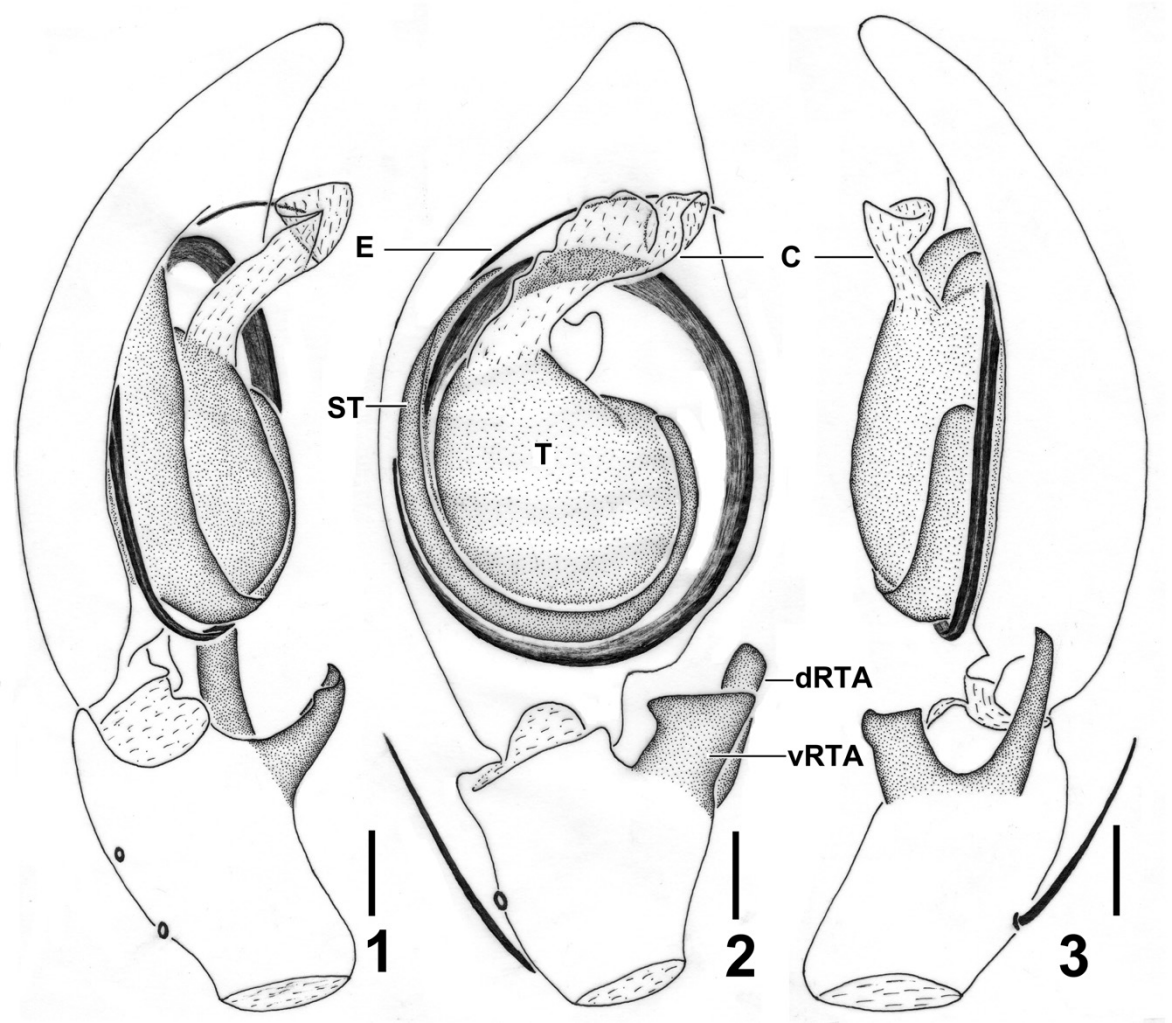
*Barylestissaaristoi* Jäger, 2008. **1–3** Left male palp (**1** prolateral **2** ventral **3** retrolateral). Abbreviations: C—conductor; dRTA—dorsal retrolateral tibial apophysis; E—embolus; ST—subtegulum; T—tegulum, vRTA—ventral retrolateral tibial apophysis. Scale bar: 0.5 mm.

**Figures 4–8.**
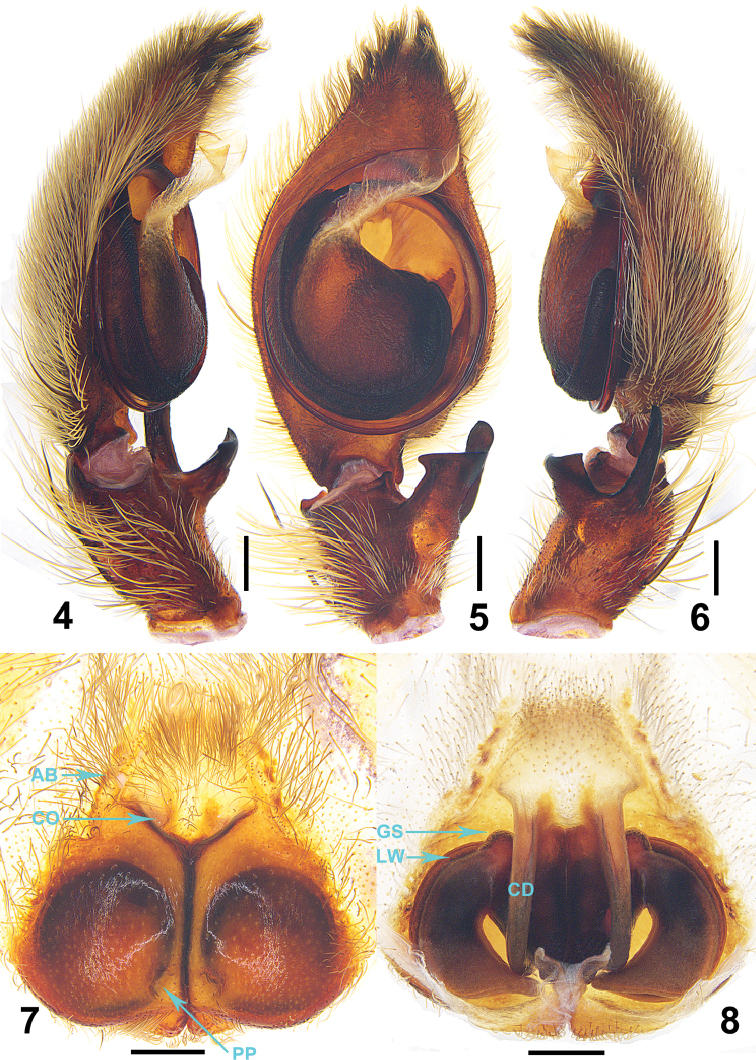
*Barylestissaaristoi* Jäger, 2008. **4–6** Left male palp (**4** prolateral **5** ventral **6** retrolateral) **7** epigyne, ventral **8** vulva, dorsal. Abbreviations: AB–anterior bands, CD–copulatory ducts, CO–copulatory opening, GS–glandular structures of internal duct system, LW–lateral winding of internal duct system, PP–posterior pits of lateral lobes. Scale bar: 0.5 mm.

###### Description.

**Figures 9–21.**
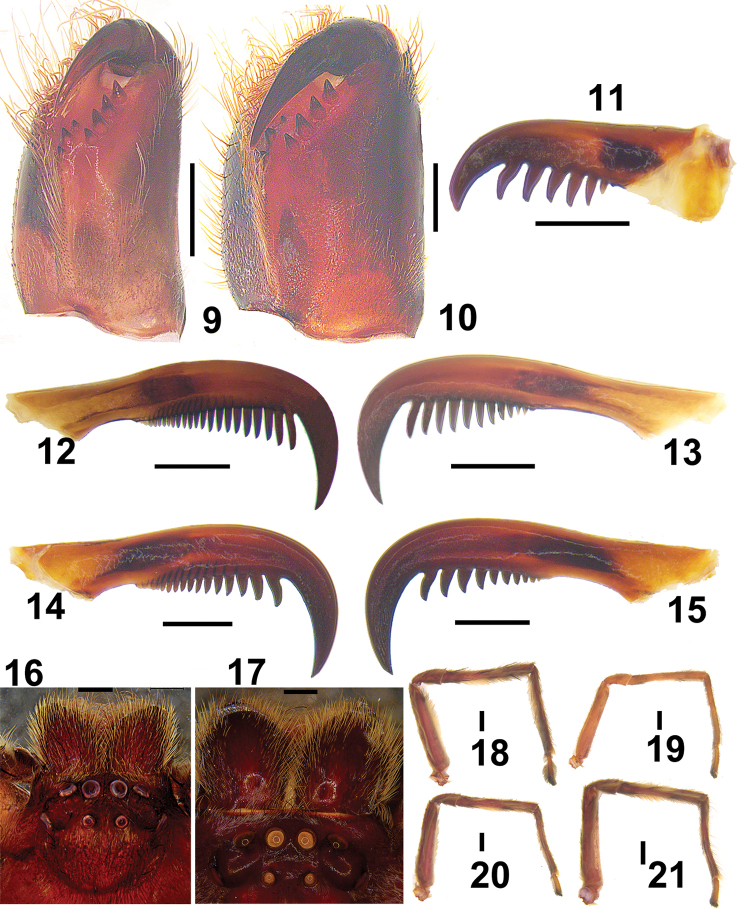
*Barylestissaaristoi* Jäger, 2008. **9, 10** Cheliceral dentition, ventral view (**9** male **10** female) **11** palpal claw of female, retrolateral view **12–15** leg I prolateral and retrolateral view of two claws (**12, 13** male **14, 15** female) **16, 17** eye arrangement, dorsal view (**16** male **17** female) **18–21** Leg I–IV (male, right). Scale bars: 1 mm (**9, 10, 16, 17**); 0.2 mm (**11–15**); 2 mm (**18–21**).

**Figures 22–25.**
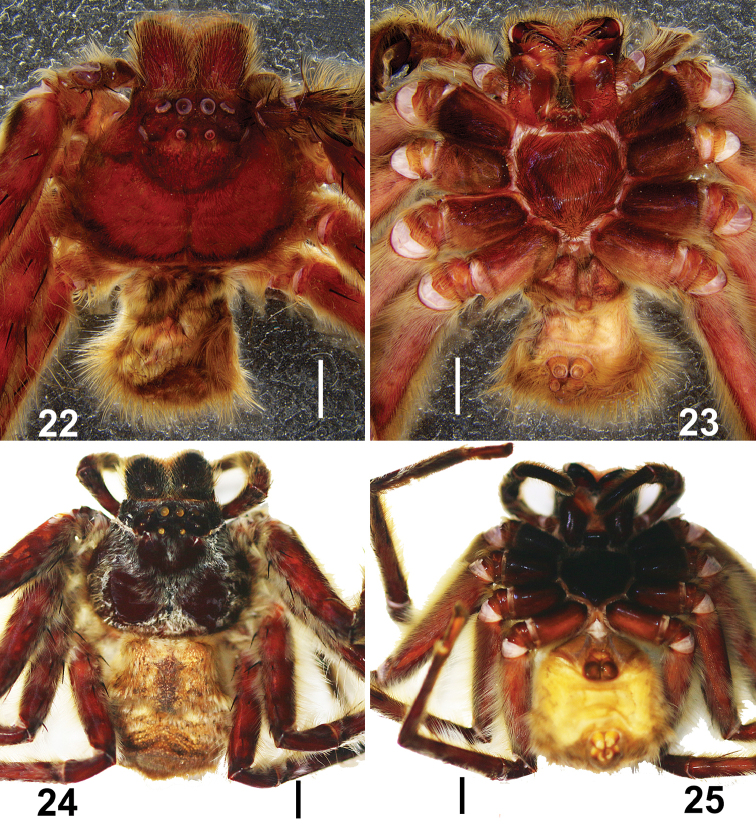
*Barylestissaaristoi* Jäger, 2008. **22, 23** Male (**22** dorsal **23** ventral) **24, 25** female (**24** dorsal **25** ventral). Scale bar: 2 mm.

***Male.***PL 6.4, PW 7.5, AW 4.0, OL 5.4, OW 3.9. Eyes: AME 0.34, ALE 0.43, PME 0.26, PLE 0.44, AME–AME 0.26, AME–ALE 0.15, PME–PME 0.59, PME–PLE 1.03, AME–PME 0.55, ALE–PLE 0.97, CHAME 0.47, CHALE0.62. Spination: Palp: 131, 101, 2021; Fe: I 333, II 000, III 333, IV 331; Pa: I 101, II 000, III 101, IV 000; Ti: I 2226, II 0004, III 2116, IV 2014; Mt: I 1014, II 0004, III 1014, IV 1016. Measurements of palp and legs: Palp 9.5 (2.9, 1.2, 1.6, –, 3.8), I 32.5 (8.6, 3.2, 8.7, 9.4, 2.6), II 27.1 (7.5, 2.3, 7.5, 7.1, 2.7), III 29.0 (9.1, 2.4, 8.4, 7.1, 2.0), IV 29.1 (9.3, 2.5, 8.1, 6.8, 2.4). Leg formula: I-IV-III-II (second leg may have fractured before collection, as it is very tiny; Figs 18–21). Cheliceral furrow with 3 anterior and 4 posterior teeth, without denticles. Claws of leg I with long and slightly curved teeth in both male and female. Female palpal claws with seven long teeth, almost same size as those of leg I (Figs 9–15). Dorsal carapace reddish-brown, posterior margins dark. Chelicerae, sternum, gnathocoxae and labium deep reddish-brown to black. Legs reddish -brown without spots and patches. Dorsal opisthosoma covered by long and dense hairs. Ventral opisthosoma uniformly yellowish-brown (Figs 22, 23). Cymbium significantly longer than tibia. Conductor membranous, arising from direction of tegulum 11:30. Embolus running 1.25 coils around tegulum, with tip situated near conductor. RTA arising medially from tibia, vRTA developed, almost rectangle-shaped and dRTA finger-shaped in ventral view (Figs 1–6).

***Female*.** For details see Jäger (2008).

###### Distribution.

**Figure 26.**
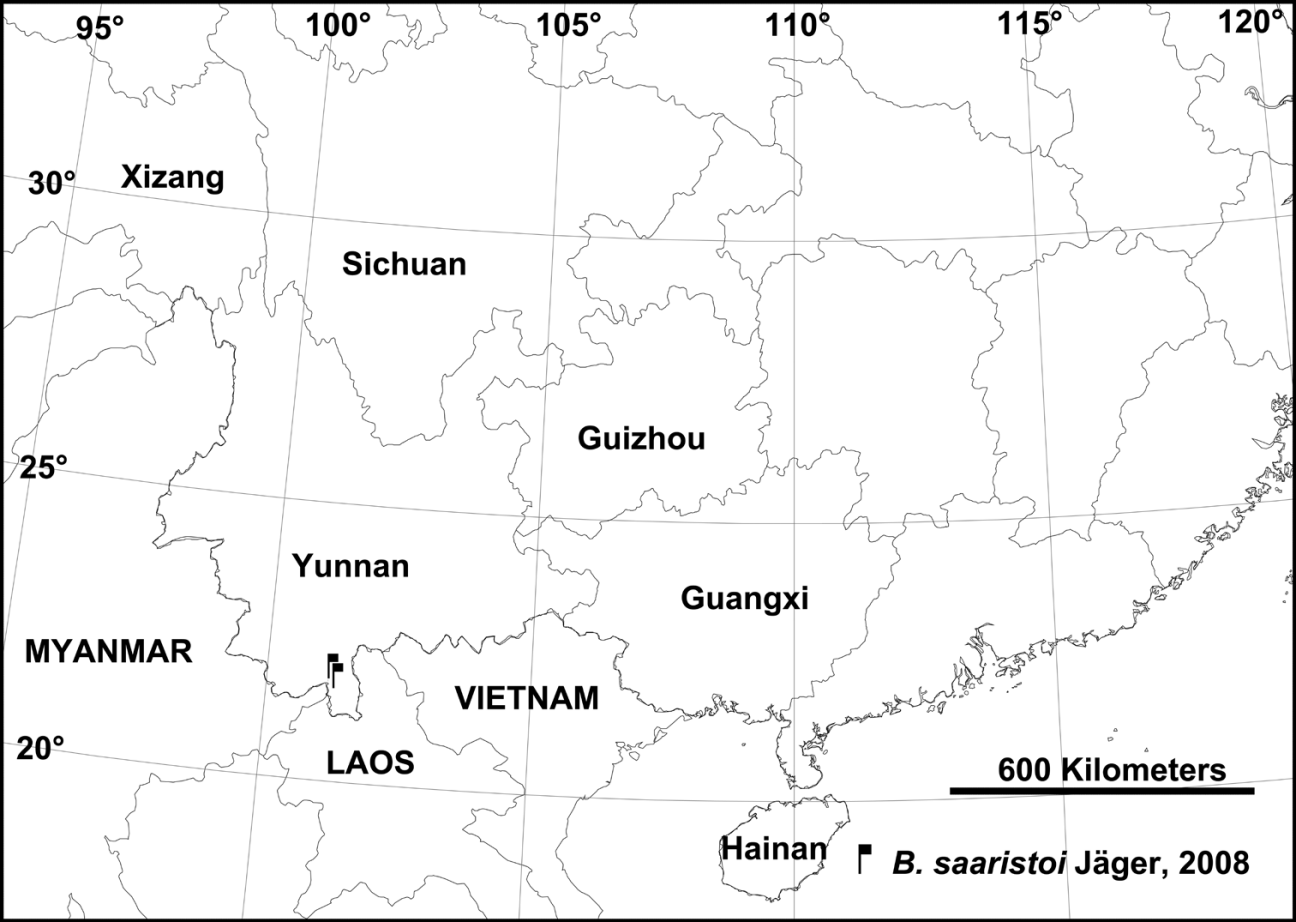
Collection localities of *Barylestissaaristoi* in Yunnan Province, China.

China (Yunnan Province, new record) (Fig. 26), Thailand (Mae Hong Son Province), Myanmar (Karen, Kayin State).

## Supplementary Material

XML Treatment for Barylestis saaristoi
